# Enhancing drug property prediction with dual-channel transfer learning based on molecular fragment

**DOI:** 10.1186/s12859-023-05413-x

**Published:** 2023-07-21

**Authors:** Yue Wu, Xinran Ni, Zhihao Wang, Weike Feng

**Affiliations:** 1 College of Traditional Chinese Medicine, Shandong University of Traditional Chinese Medicine, Jinan, China; 2 College of Pharmacy, Shandong University of Traditional Chinese Medicine, Jinan, China; 3 College of Intelligence and Information Engineering, Shandong University of Traditional Chinese Medicine, Jinan, China

**Keywords:** Drug property prediction, Transfer learning, Molecular representation learning

## Abstract

**Background:**

Accurate prediction of molecular property holds significance in contemporary drug discovery and medical research. Recent advances in AI-driven molecular property prediction have shown promising results. Due to the costly annotation of in vitro and in vivo experiments, transfer learning paradigm has been gaining momentum in extracting general self-supervised information to facilitate neural network learning. However, prior pretraining strategies have overlooked the necessity of explicitly incorporating domain knowledge, especially the molecular fragments, into model design, resulting in the under-exploration of the molecular semantic space.

**Results:**

We propose an effective model with FRagment-based dual-channEL pretraining (FREL). Equipped with molecular fragments, FREL comprehensively employs masked autoencoder and contrastive learning to learn intra- and inter-molecule agreement, respectively. We further conduct extensive experiments on ten public datasets to demonstrate its superiority over state-of-the-art models. Further investigations and interpretations manifest the underlying relationship between molecular representations and molecular properties.

**Conclusions:**

Our proposed model FREL achieves state-of-the-art performance on the benchmark datasets, emphasizing the importance of incorporating molecular fragments into model design. The expressiveness of learned molecular representations is also investigated by visualization and correlation analysis. Case studies indicate that the learned molecular representations better capture the drug property variation and fragment semantics.

**Supplementary information:**

The online version contains supplementary material available at 10.1186/s12859-023-05413-x.

## Introduction

One of the most foundational and crucial tasks in the domain of drug discovery pertains to the accurate prediction of molecular properties. Compared with conventional in vitro and in vivo experiments, computational methods have the potential to expedite the overall process of identifying better drug candidates with specific characteristics [1, 2]. In general, the performance of molecular property prediction is mainly affected by two stages. The initial stage involves molecular featurization design [3–5], which aims to translate chemical information into structured data recognizable by machine learning algorithms. The subsequent stage, known as molecular representation learning [6–8], focuses on the development of methods for representing molecules as numerical vectors that encapsulate rich semantic biochemical information, either through manual [9] or automatic means [10]. Our paper, situated within the second stage, delves into self-supervised molecular representation learning techniques that implicitly extract biomedical domain knowledge via drug molecular fragments.

Due to the inherent benefits of graphs in representing molecules, graph-based models, ranging from convolutional [11] to spatial neural networks [12, 13], have garnered attention in initial efforts towards supervised molecular representation learning. However, it is hampered by the lack of labeled property [14] and the out-of-distribution problem [10, 15], which have spurred the development of transfer learning approaches. A common framework involves pretraining the model with proxy tasks on extensive unlabeled molecular datasets, followed by fine-tuning the learned model on labeled downstream tasks. Prior studies [16–20] employ various augmentation methods to construct molecular view pairs for contrastive learning, maximizing the agreement between different augmented views. Some models, on the other hand, use generative learning [21] to reconstruct partial information of the sample itself [22, 23], enabling the model to learn the molecular semantic space.

Despite of some encouraging headway, most of the prior studies tend to overlooked the potential benefits of incorporating domain knowledge into model architecture, which can explicitly integrate biochemical information into model training. In the domain of pharmaceuticals, molecular fragments are of vital importance in determining molecular properties. For example, adrenergic receptor agonists with catechol structure (catechol hydroxyl group) are easily decomposed by COMT (catechol O-methyltransferase) in vivo, with poor stability and short action time, which affects the effectiveness of the drug. In comparison, adrenergic receptor agonists with non-catechol structure have much stronger stability [24]. Moreover, we further present a exploratory experiment to verify the feasibility and effectiveness of fragment-based model design in the Additional file 1.

**Fig. 1:**
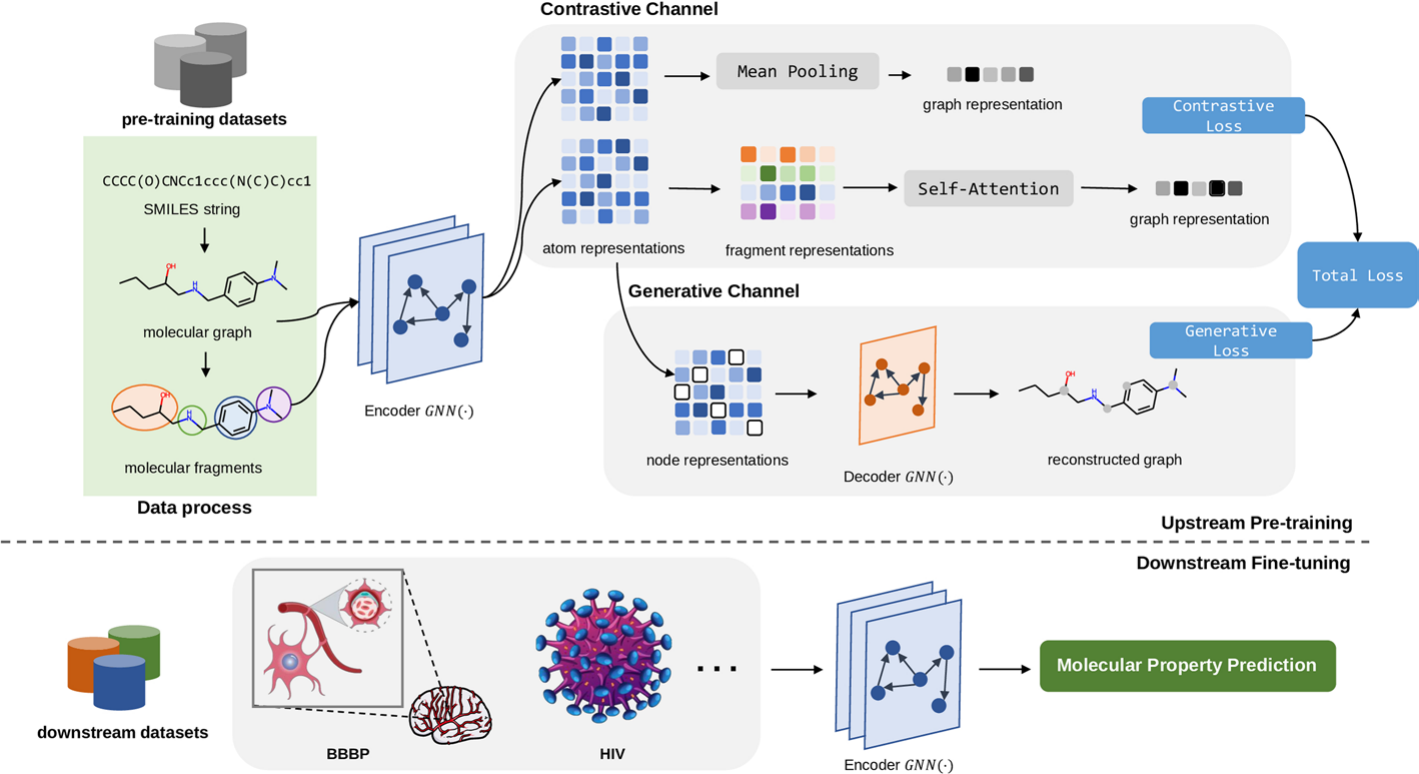
The proposed FREL model. In the pre-training phase, the GNN encoder takes molecular graph and fragments as inputs, which are respectively fed into the subsequent contrastive channel and generative channel. The model parameters are optimized with the sum of contrastive loss and generative loss to learn intra- and inter-molecule agreement. We express our gratitude for the use of the illustration of the blood-brain barrier and HIV virus, which were obtained from the websites https://smart.servier.com/ and https://www.vecteezy.com/, respectively. We confirm that permission was given to reproduce these works

Motivated by intuitive inspiration and exploratory experiment, we propose a novel and effective framework with FRagment-based dual-channEL pretraining (FREL), that comprehensively employs generative learning and contrastive learning to achieve intra- and inter-molecular agreement, respectively. The overall framework is demonstrated in Fig. 1. Specifically, for the contrastive learning channel, we generate two correlated molecular views of the same molecule. Then, we define a contrastive loss to maximize the inter-molecular agreement. For generative learning channel, we randomly mask partial node features and leverage a decoder to reconstruct the masked features based on intra-fragment information. By combining aforementioned contrastive and generative loss, FREL is expected to learn both intra- and inter-molecular agreement. We further support the effectiveness of our approach with theoretical analysis from the perspective of information theory.

We evaluate the performance of our FREL model on 10 widely-used benchmark datasets from MoleculeNet [10] and malaria [25] that cover a wide range of molecular property prediction tasks, including classification and regression. The results reveal that FREL improves non-pretraining baselines without negative transfer and achieve the state-of-the-art (SOTA) performance. Moreover, we conduct extensive experiments to evaluate the expressiveness of molecular representations by visualization and statistical methods. The main contributions of this work are three-fold:

- With intuitive biochemical inspiration and convincing exploratory experiment, we rethink the necessity to incorporate molecular fragment into model design in molecular property prediction tasks.
- We propose a novel dual-channel self-supervised pretraining strategy to learn intra- and inter-molecule agreement, enabling effective molecular representation learning.
- We evaluate our method on extensive molecular property prediction tasks. Experimental results demonstrate that FREL achieve superior performance compared with competitive baselines. Further experiments investigate the expressiveness of learned molecular representation.

## Related work

Molecular pre-training, achieved through self-supervised training on massive amounts of unlabeled upstream data, enables models to capture rich semantic information about molecules. This research paradigm has demonstrated its effectiveness in enhancing predictive performance on downstream tasks. In the context of graph-based molecular pre-training, early works focused on adapting classical graph self-supervised methods to molecular graph training. Methods such as AttrMask, ContextPred [16], GPT-GNN [26], and GraphMAE [27] utilize generative learning to predict masked features within the molecule, allowing for the capture of structural and semantic properties of the graph. On the other hand, GraphCL [17], JOAO [28], and MolCLR [29] leverage contrastive learning to provide supervision signals during model training. Additionally, some methods approach the problem from the perspective of mutual information or clustering to capture cross-level graph semantics.

However, traditional graph self-supervised learning often overlook the incorporation of domain-specific knowledge in the molecular domain, which can impact the positive transfer brought by pre-training. In recent years, tailored pre-training strategies specific to molecular graphs have been proposed, broadly categorized into atom-level strategies and fragment-level strategies. GraphMVP [20], 3D Infomax [30], GeoSSL [31], and GEM [32] enhance the model’s ability to capture energy information by utilizing the atomic coordinates of the molecular 3D conformation as auxiliary inputs. Mole-BERT [33], on the other hand, explores the feasibility of masked atom modeling from an optimized atomic encoding perspective. Given the crucial role of functional group information in determining molecular properties, a subset of concurrent works has focused on mining functional group or fragment information. For instance, methods like GROVER [34], MGSSL [35], and iMolCLR [36] explicitly incorporate chemical priors at the pre-training stage from the perspective of motifs or fragments. In recent years, low-data drug discovery and the few-shot setting have gained increasing attention, aiming to address the challenges of limited labeled data and out-of-distribution generalization in downstream tasks [37, 38].

However, prior work has not adequately integrated unsupervised training strategies with the introduction of domain-specific knowledge (such as molecular fragments), which hinders exploration of intra- and inter-molecular semantics. Therefore, we aim to explore the possibilities for a more integrated approach in this regard.

## Results

In this section, we present empirical evaluation of our proposed FREL model and demonstrate its effectiveness. Specifically, the experiments aim to investigate the following research questions.

- **RQ1 (Overall performance).** Does the proposed model bring positive transfer and outperform state-of-the-arts on molecular property prediction tasks?
- **RQ2 (Representation expressiveness).** How expressive are pre-trained molecular representations?
- **RQ3 (Ablation studies).** How do the different channels contribute to the model performance?
- **RQ4 (Sensitivity Analysis).** How does different hyperparameters affect model performance?

We first provide a brief introduction of the experimental configurations, with more detailed settings available in the Methods section. We then demonstrate the performance of our proposed FREL on various property prediction tasks. Additionally, we leverage visualization and case studies to better showcase the superiority of the learned representations. Lastly, we present the results of ablation experiments and sensitivity analysis.

### Experimental configurations

#### Datasets and baselines

We choose GEOM-Drugs [39] as the pre-training dataset following GraphMVP [20] and evaluate the pre-trained model on ten downstream datasets: BBBP [40], Tox21 [41], ToxCast [42], SIDER [43], MUV [44], HIV [45], BACE [46], ESOL [47], Lipophilicity [48] and Malaria [25].

For comprehensive comparison, we select the following two groups of Self-Supervised Learning (SSL) methods as primary baselines in our experiments.

- Generic graph SSL models: AttrMask, ContextPred [16], InfoGraph [49], GPT-GNN [26], GraphLoG [18], GraphCL [17], JOAO [28], and GraphMAE [27].
- Molecular SSL models: GROVER-Contextual (GROVER-C), GROVER-Motif (GROVER-M) [34], MGSSL [35], GraphMVP [20] and Mole-BERT [33].

#### Evaluation protocols

We evaluate the performance of our model differently depending on the task. For classification tasks, we use the Area Under the Receiver Operating Characteristic curve (ROC-AUC) as the performance metric, where higher values indicate better performance. For regression tasks, we use Root Mean Squared Error (RMSE) as the performance metric, where lower values indicate better performance. The TPR, FPR and RMSE are formalized as follow:

1 $$\begin{aligned} \text {FPR}&= \frac{\text {False Positive}}{\text {False Positive} + \text {True Negative}} \end{aligned}$$

2 $$\begin{aligned} \text {TPR}&= \frac{\text {True Positive}}{\text {False Positive} + \text {True Negative}} \end{aligned}$$

3 $$\begin{aligned} \text {RMSE}&= \sqrt{\frac{1}{n} \sum _{i=1}^{n} (y_i - \hat{y_i})^2} \end{aligned}$$

To ensure the robustness of our results, we report the averaged performance with the standard deviation by repeating each experiment using three different random seeds under scaffold splitting, following previous work [20].

### Main results on molecular property classification

The performance of molecular property prediction tasks is summarized in Table 1. Our model exhibit outstanding performance on seven classification datasets for molecular property prediction, outperforming most of the baseline models. Specifically, our model achieve the state-of-the-art results on five of the seven datasets, and comparable results on the remaining two. On average, our model exhibit superior performance to all baseline models, with a 0.9% improvement over the second-best performing model.

**Table 1:** Results for seven molecule property prediction tasks in terms of ROC-AUC (%, $\uparrow$)

Pretraining	BBBP	Tox21	ToxCast	SIDER	MUV	HIV	BACE	Avg.
–	65.4±2.4	74.9±0.8	61.6±1.2	57.7±2.4	71.0±2.5	75.3±0.5	72.6±4.9	68.36
EdgePred	64.5±3.1	74.5±0.4	60.8±0.5	56.7±0.1	73.3±1.6	75.1±0.8	64.6±4.7	67.07
AttrMask	70.2±0.5	74.2±0.8	62.5±0.4	60.4±0.6	73.9±1.3	74.3±1.3	77.2±1.4	70.39
GPT-GNN	64.5±1.1	75.3±0.5	62.2±0.1	57.5±4.2	76.1±2.3	75.1±0.2	77.6±0.5	69.76
InfoGraph	69.2±0.8	73.0±0.7	62.0±0.3	59.2±0.2	74.0±1.5	74.5±1.8	73.9±2.5	69.40
ContextPred	**71.2±0.9**	73.3±0.5	62.8±0.3	59.3±1.4	72.5±2.2	75.8±1.1	78.6±1.4	70.50
GraphLoG	67.8±1.7	73.0±0.3	62.2±0.4	57.4±2.3	73.1±1.7	73.4±0.6	78.8±0.7	69.39
GROVER-C	70.3±1.6	75.2±0.3	62.6±0.3	58.4±0.6	72.3±0.9	75.9±0.9	*79.2±0.3*	70.56
GROVER-M	66.4±3.4	73.2±0.8	62.6±0.5	60.6±1.1	73.3±2.0	73.8±1.4	73.4±4.0	69.04
GraphCL	67.5±3.3	75.0±0.3	62.8±0.2	60.1±1.3	77.1±1.0	75.0±0.4	68.7±7.8	69.46
JOAO	66.0±0.6	74.4±0.7	62.7±0.6	60.7±1.0	77.0±2.2	76.6±0.5	72.9±2.0	70.04
GraphMVP	68.5±0.2	74.5±0.4	62.7±0.1	60.3±1.6	75.0±1.4	74.8±1.4	76.8±1.1	70.37
GraphMAE	70.3±0.9	75.0±0.4	62.9±0.3	59.9±0.5	76.9±2.6	*76.7±0.9*	75.4±1.4	71.19
MGSSL	67.8±0.7	75.1±0.3	62.6±0.4	60.7±0.8	75.5±2.1	75.2±0.9	76.9±1.1	70.54
Mole-BERT	**71.2**±**1.5**	*75.5±0.6*	*63.9±0.3*	**61.5±0.5**	*77.1±1.6*	76.5±1.1	78.8±1.5	*72.07*
FREL	*70.8±0.8*	**75.8±0.4**	**64.9±0.8**	*60.9±0.6*	**78.9**±**1.2**	**77.8**±**0.5**	**80.3**±**0.3**	**72.77**

We highlight the best- and the second-best performing results in boldface and italicized, respectively

We make other observations as follows. Firstly, compared with randomly initialized baseline, FREL obtains more accurate and robust predictions, indicating that our pretraining framework can capture useful knowledge from large, unlabeled datasets and migrate the learned semantics to downstream tasks without negative transfer. Secondly, we can observe that prior work has already achieved promising performance, especially in scaffold split settings. For example, Mole-BERT, as the current SOTA methods, only obtains a 1.2% absolute improvement over its best baseline GraphMAE in terms of average ROC-AUC. Our model expands the limits of performance without extensive hyperparameter tuning, achieving an absolute improvement of up to 0.9% in terms of average ROC-AUC over Mole-BERT. Lastly, it is shown tha second-best models often fail to achieve robust performance gains on most datasets, which can be attributed to the diversity of downstream drug properties. For instance, although GROVER-C achieved second-best performance on the BACE dataset, it exhibits poor performance on the MUV dataset. In contrast, our proposed framework achieve robust performance on diverse drug property prediction tasks, highlighting the robustness of FREL.

### Results on molecular property regression

**Fig. 2:**
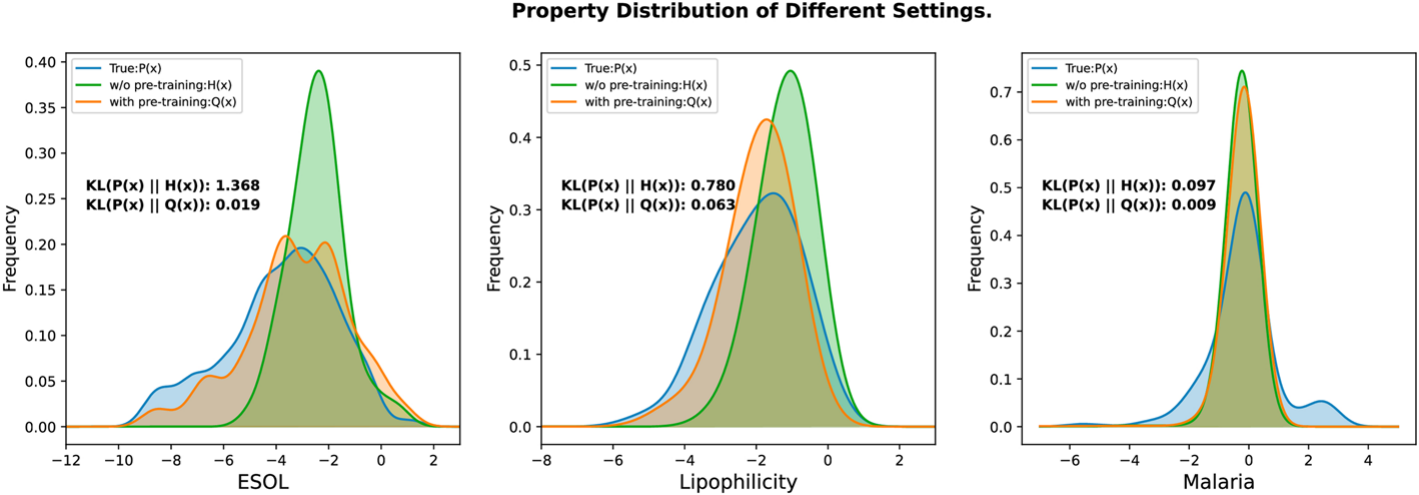
Distribution of the predicted property and true label

To further demonstrate the effectiveness of our proposed model FREL, we further conduct experiments on molecular property regression on the ESOL [47], Lipophilicity [48] and Malaria dataset [25]. Table 2 presents the performance comparison of FREL with one non-pretraining baselines and five state-of-the-art pretraining baselines AttrMask, ContextPred [16], JOAO [28], GraphMVP [20] and GraphMAE [27]. Furthermore, we conduct a comparison between the predicted value distributions obtained from pre-trained and non-pre-trained models, and the true label distribution. The discrepancy between the predicted value distribution and the true label distribution is measured by KL divergence, as shown in Fig. 2

**Table 2:** Additional results on four molecular property regression tasks in terms of Root-Mean-Square Error (RMSE, $\downarrow$)

Pretraining	ESOL	Lipophilicity	Malaria	Avg.
—	1.361±0.016	0.797±0.006	1.122±0.011	1.093
AttrMask	1.115±0.048	0.791±0.004	1.119±0.014	1.008
ContextPred	1.199±0.037	0.763±0.020	1.101±0.015	1.021
JOAO	1.123±0.019	0.769±0.007	1.145±0.010	1.012
GraphMAE	1.282± 0.023	0.769± 0.003	1.098± 0.012	1.050
GraphMVP	1.094±0.021	0.776±0.016	1.114±0.013	0.995
FREL	**1.088**±**0.034**	**0.744**±**0.001**	**1.087**±**0.009**	**0.973**

The lowest prediction error is highlighted in boldface

It is seen from Table 2 that our FREL model also obtains positive transfer compared with non-pretraining baseline and achieves the SOTA performance on all datasets. Moreover, it can be observed from Fig. 2 that the predicted molecular properties by the pre-trained model show a better match with the true label distribution of drug properties compared with non-pre-trained one, which also verifies the effectiveness of our framework and demonstrates the importance of taking into consideration the molecular fragments.

### Interpretation and analysis

Although we have incorporated information on molecular fragments through explicit model design, the black-box nature of deep learning still hinders our understanding of the specific reasons behind performance improvements. Based on the experimental results mentioned above, we will attempt to provide some hypotheses for performance improvements based on the characteristics of the specific dataset.

Note that most of the datasets we used for downstream tasks are ADMET property prediction tasks: chemical Absorption (A), Distribution (D), Metabolism (M), Excretion (E), and Toxicity (T), and we thus group the ten end tasks according to their prediction targets in the following analysis. We provide detailed analysis as follows:

- Tox21, Toxcast, and Sider are datasets that provide information on chemical toxicity, toxicological assessment, and side effects and adverse reactions (ADR) of listed drugs. These datasets are relevant to the study of drug toxicity, specifically its side effects. Our research suggests that the success of these datasets can be attributed to the identification of structural alerts and toxicophores, such as aromatics and nitro groups, which are associated with hepatotoxicity. These functional groups are essential components of drugs and cannot be easily replaced. [50].
- The blood-brain barrier (BBB) is a highly selective interface between the circulating blood and the brain extracellular fluid, which serves to protect the brain from potentially harmful foreign substances present in the bloodstream. BBBP refers to the permeability of the BBB, which is determined by several factors such as the size of the molecule, the expression of relevant transporters and enzymes, and lipid/water solubility. While the functional groups of a substance may affect its lipid/water solubility, they cannot directly alter the permeability of the BBB. As a result, the impact on the permeability of the BBB is limited, which may explain why the outcomes of our experiment were not as optimal as we had hoped. [51].
- Drug molecular fragments play a significant role in the inhibition of HIV-1 replication, where the same functional groups have similar antiviral activity. Take tenofovir for example, and a series of aryl phenoxy-amidate derivatives of it, showed potent activities against the replications of HIV-1 [52]. Furthermore, the modification of functional groups during the transformation of precursor compounds into anti-HIV drugs is an effective strategy for detecting precursor compounds.
- The ESOL and Lipophilicit datasets describe the hydrophilicity and lipophilicity of drug molecules, the ratio of a drug to its lipid/water distribution. Compared with macromolecules, the larger the molecular weight, the more lipophilic. Since most molecules in production and life are smaller, the lipid/water solubility of molecules is mainly affected by functional groups. For example, the presence of -COOH significantly increases the hydrophilicity of a molecule. Therefore, our model achieves better performance [47].
- Malaria is a data set on antimalarial drug inhibitors collected by GlaxoSmithKline ( GSK ). This data set discloses the structure of effective, drug-like antimalarial compounds in the hope of finding the key to new malaria treatment. These compounds exhibit higher molecular weight and hydrophobicity index compared to other compounds. We speculate that the success of this experiment may be attributed to these characteristics of the compounds in the dataset.

### Investigation on molecular representation

**Fig. 3:**
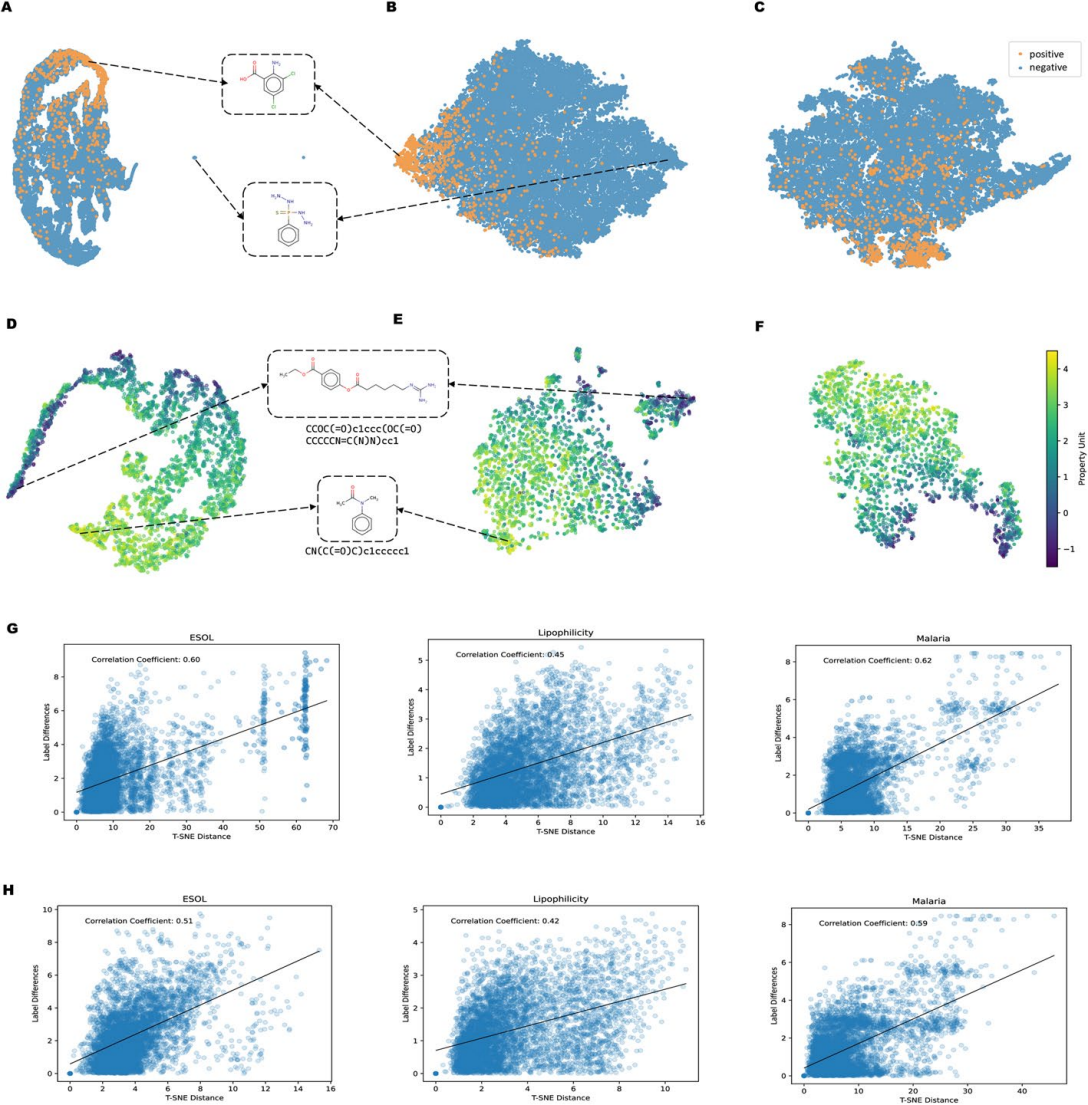
Investigations on molecular representation based on t-SNE analysis. **A**, t-SNE analysis with random initialized GIN model on HIV dataset. **B**, t-SNE analysis with model pretrained by FREL on HIV dataset. **C**, t-SNE analysis with model pretrained by GraphMAE on HIV dataset. **D**, t-SNE analysis with random initialized GIN model on Lipophilicity dataset. **E**, t-SNE analysis with model pretrained by FREL on Lipophilicity dataset. **F**, t-SNE analysis with model pretrained by GraphMAE on Lipophilicity dataset. **G**, correlation analysis of t-SNE distance and label difference on regression tasks pretrained by FREL. **H**, correlation analysis of t-SNE distance and label difference on regression tasks pretrained by GraphMAE

We use t-SNE (t-distributed Stochastic Neighbor Embedding) [53] to intuitively show the molecular representation learned by FREL. The t-SNE algorithm is a dimensionality reduction technique that is commonly used for visualizing high-dimensional data in a 2D space. Points that are similar in the high-dimensional space are mapped to nearby points in the low-dimensional space, while points that are dissimilar are mapped to distant points. As shown in Fig. 3, we perform t-SNE analysis on the HIV and Lipophilicity datasets to compare the superiority of our pre-training strategy against non-pre-trained models. We further visualize the t-SNE plots of the SOTA atom-level pre-training strategy, GraphMAE, to highlight the advantages of considering molecular fragments. Distinct class labels are represented via different colors. It is worth noting that the visualized representations are fine-tuned by downstream labels.

After pre-training, the representations obtained by the model exhibit clustering characteristics, with similar labeled representations being closer to each other, and representations with dissimilar labels being distributed further apart, thereby enhancing the discriminability of molecules belonging to different categories. This is beneficial for improving the performance during downstream classification and regression. In contrast, the non-pre-trained model performs poorly in this regard. As shown in figure **A** and **D** from Fig. 3, representations with distinct labels are closer to each other. Additionally, we present four examples with clearly distinct properties for each dataset. Taking the Lipophilicity dataset as an example, the molecule at the top has poor hydrophobicity due to the presence of amino groups, whereas the molecule at the bottom consists of nonpolar functional groups such as phenyl group and alkyl groups that enhance lipophilicity. This is consistent with our motivation for designing the model based on molecular fragment.

To better explore the expressiveness of the learned representation obtained from model after pre-training, we draw the scatter plots of the distance between the molecular representations after t-SNE dimensionality reduction and the absolute value distance between their true labels on three regression datasets, as illustrated in figure G and H from Fig. 3. The horizontal axis represents the Euclidean distance between the molecular representations after t-SNE dimensionality reduction, and the vertical axis represents the absolute difference between the molecular labels. The correlation coefficient is displayed in the upper left corner of each plot, showing a positive correlation between the t-SNE distance and label difference, indicating that the pre-trained representation can better capture features affecting property variation. It can reflect the effectiveness of the learned representations and provide a more intuitive understanding. While GraphMAE produce better representations compared to non-pre-trained model, it demonstrate suboptimal ability in capturing label variation and molecular separability compared to our approach. This highlights the necessity of incorporating fragment information in our proposed pretraining strategy.

**Fig. 4:**
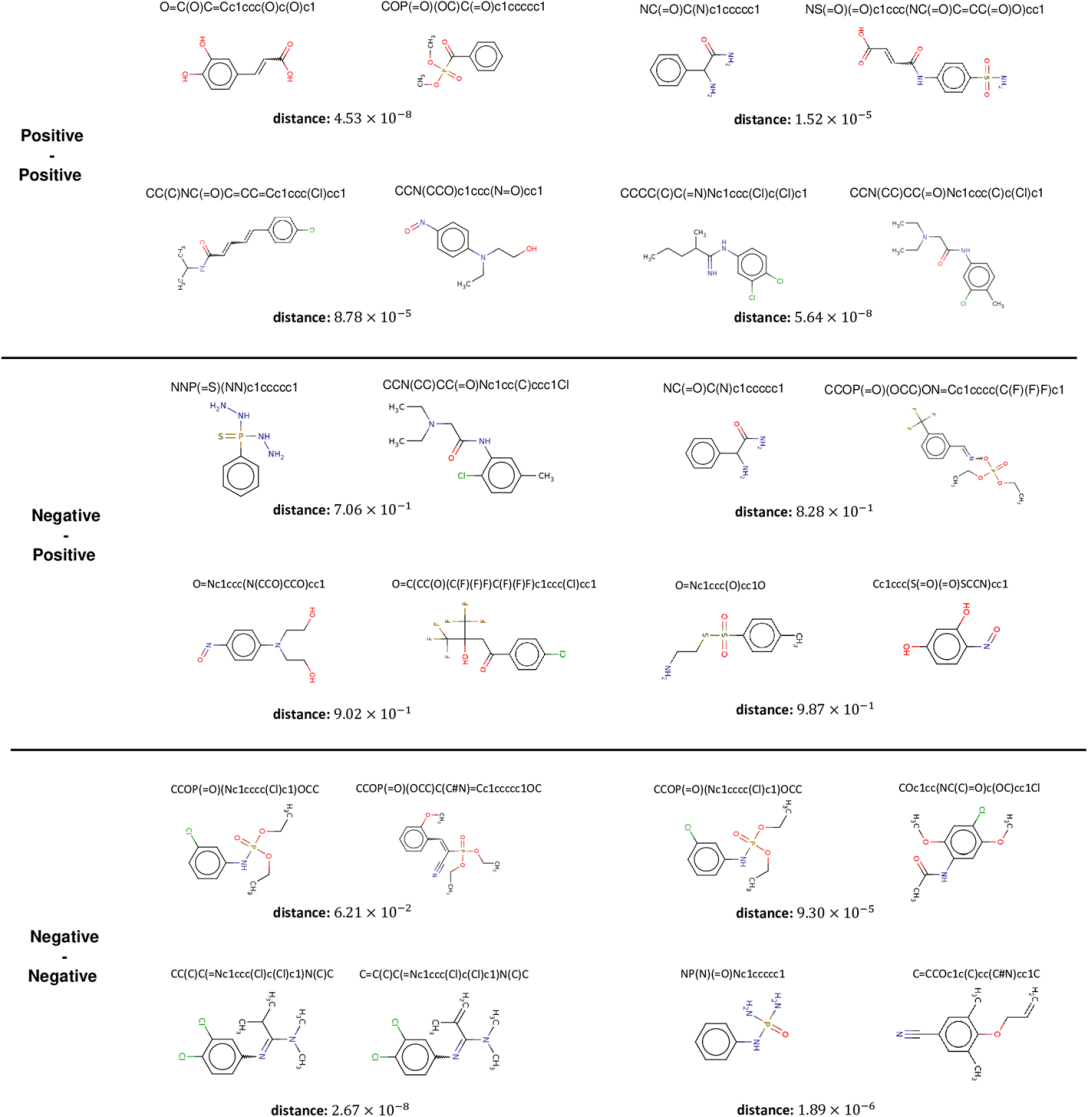
Case study of learned molecular representations

Based on the aforementioned correlation analysis, we conduct a case study on the HIV dataset to further verify the relationship between the distance learned between representations and the difference in molecular properties. Since the HIV dataset evaluates whether molecular compounds are able to inhibit HIV replication (positive or negative), we randomly selected three sets of molecular instances for observation, namely positive-positive, positive–negative, and negative-negative. The results are shown in Fig. 4.

It can be observed that representations of molecules with the same properties are generally closer to each other, with distances ranging from $10^{-8}$ to $10^{-5}$. On the other hand, molecules with different properties have larger distances, with most distances ranging from $10^{-2}$ to $10^{-1}$. This indicates that our pre-training strategy can help the model learn better molecular semantic information, thus facilitating the completion of downstream tasks.

### Empirical analysis of capturing fragment semantics

To support our key motivation and justify that FREL enables better learning of fragment semantics compared to other atom-based pretraining strategies, we further conduct two experiments, including (1) *Aromatic Ring Counting* and (2) *Hydroxyl-Containing Molecules Solubility Regression*. The description of experimental settings are as follow:

- For aromatic ring counting, we randomly draw 10K molecules from GEOM-Drugs dataset to test whether model pretrained with different strategies can recognize the number of aromatic rings of each molecule, which is an informative descriptor determining various drug properties [54]. The performance in terms of Mean Average Error (MAE) are shown in Table 3.
- For the hydroxyl-containing molecules solubility regression, we select all molecules with hydroxyl groups from the ESOL dataset. We then perform a regression task on their water solubility using the same experimental setup as described in the Experimental Configurations. The performance in terms of Rooted Mean Squared Error(RMSE) are shown in Table 3.

**Table 3:** The results of case studies (Ring Counting & Solubility Regression)

	Random	AttrMask	GraphCL	GraphMVP	MGSSL	FREL
Ring Count (MAE, $\downarrow$)	0.1949	0.1373	0.1222	0.1207	0.1154	**0.0956**
Solubility (RMSE, $\downarrow$)	1.4982	1.2655	1.1877	1.1620	1.1989	**1.0652**

The best performing result is highlighted in bold

Given the intimate relationship between the experiments we proposed and molecular fragment information, the performance of these experiments can serve as an indicator of the capability to capture molecular fragment semantics. A superior performance signifies a stronger proficiency in capturing the semantic of molecular fragments. Based on the results from Table 3, we observe that our proposed pretraining strategy achieves the best performance compared to other atom-level and fragment-level methods, indicating its superiority in capturing molecular fragment semantics.

### Ablation studies and sensitivity analysis

To answer RQ3, we conduct ablation studies on the effect of different pretraining channels to verify that different modules of FREL can independently provide beneficial impact. We consider the following model variants for further inspection. Except the modifications in specific modules, other implementations remain the same as previously described.

- FREL **–**${\mathcal {C}}$ removes the generative learning channel in the pretraining phase and simply uses the ${\mathcal {L}}_{{\mathcal {C}}}$ in Eq. 10 as the pretraining objective.
- FREL **–**${\mathcal {G}}$ modifies the pretraining objective by removing the contrastive learning channel and uses the ${\mathcal {L}}_{{\mathcal {G}}}$ in Eq. 10 as the pretraining objective.

We report the performance of model variants in Additional file 2. It is seen that all three variants achieve downgraded performance, which empirically rationalizes the design choice of our molecular pretraining framework with dual-channel pretraining. Specifically, the performance of FREL–${\mathcal {C}}$ and FREL–${\mathcal {G}}$ is inferior to that of FREL, demonstrating the necessity of combining self-supervised information from both channels. In addition, FREL–${\mathcal {G}}$ occasionally obtains better performance than the FREL–${\mathcal {C}}$. For example, on the MUV dataset, FREL–${\mathcal {G}}$ achieves 2.1% improvement in terms of ROC-AUC. It indicates that the proposed generative learning strategy is more effective compared with the contrastive learning.

Moreover, we further evaluate the performance of FREL with respect to two model-specific hyper-parameters: the mask ratio *m* and temperature coefficient $\tau$. Intuitively, a small mask ratio is simpler for reconstruction, but it can lead to the inability to capture effective molecular semantic information. Conversely, a large mask ratio can result in a reduction of available information, lacking sufficient self-supervision signals. Therefore, we select different candidate values at equal intervals of 10% within the range of 10% to 60%. For the temperature coefficient, we also select six candidate values [0.01, 0.07, 0.1, 0.3, 0.5, 0.7] for analysis. To demonstrate the joint influence of these two key hyper-parameters, we use an enumeration combination of the candidate values for both parameters and obtain the results shown as Additional file 3.

## Discussion and future work

Prior works on drug property prediction have two main limitations. Firstly, they tend to overlook the effectiveness of explicitly incorporating molecular fragment information into model design, despite the crucial role that molecular fragments play in determining molecular properties. Secondly, the effective utilization of both intra- and inter-molecular relationships for self-supervised pretraining has not been adequately addressed, resulting in suboptimal molecular representation learning.

Our model has demonstrated the ability to partially mitigate the aforementioned issues and yield performance improvements. However, there are still some limitations to address. Currently, most molecular pre-training models are based on the GIN encoder, which tends to have a smaller parameter count due to challenges such as over-smoothing [55, 56] and over-squashing. In contrast, in the fields of NLP and CV, larger parameter sizes have shown significant benefits for pre-training and bring more positive transfer. Therefore, an intriguing avenue for future research lies in exploring how existing pre-training strategies can be better adapted to models with larger capacities.

Furthermore, the method of fragmenting molecules based on BRICS decomposition is rather crude, while the approach of decomposing molecules into functional groups based on chemical definitions is overly meticulous, thus limiting effective exploration of the chemical semantic space. Consequently, an unresolved issue is how to identify valuable molecular subgraphs for pre-training strategies. In the future, we aim to find effective solutions to these open-ended questions that can drive drug discovery forward.

## Conclusions

Pretraining methods have emerged as a prominent research focus in the field of drug property prediction. Nevertheless, prevalent pretraining methods in the field often lack explicit incorporation of biochemical knowledge and exhibit a limited scope in the design of self-supervised strategies. To this end, we propose a novel framework, coined FREL, which comprehensively employs generative learning and contrastive learning to achieve intra- and inter-molecule agreement, respectively. Our approach explicitly integrates molecular fragment information into the model design. We evaluate the effectiveness of FREL on ten benchmark datasets and achieve the state-of-the-art performance. Further empirical analysis supports our key motivation that molecular fragment has the potential to boost the performance of drug property prediction. Overall, our work highlights the necessity of incorporating molecular fragments into model design and provides a promising solution for drug property prediction task.

## Methods

### Preliminaries

We begin by introducing some common notations for Graph Neural Networks (GNNs) and outlying the key concepts used in this work. Each molecule can be represented as an undirected graph, with atoms as nodes and chemical bonds as edges. Let ${\mathcal {G}}=({\mathcal {V}},{\mathcal {E}})$ denote a molecule, where $v \in {\mathcal {V}}$ represents atom and $(u,v) \in {\mathcal {E}}$ represents chemical bond connecting atom *u* and *v*. The feature of node *v* and edge (*u*, *v*) with *D* dimension are denoted as $\varvec{x}_{u}, \varvec{x}_{uv} \in {\mathbb {R}}^{D}$, respectively. Graph neural networks are message-passing networks. Formally, given a node *v*, its representation vector $\varvec{h}_{v}^{(k)}$ at the *k*-th layer is formalized by

4 $$\begin{aligned} \begin{aligned} \varvec{a}_v^{(k)}&= \text {AGGREGATE}^{(k)}\left( \left\{ \varvec{h}_{v}^{(k-1)},\varvec{h}_{u}^{(k)},\varvec{x}_{uv}|u \in {\mathcal {N}}(v) \right\} \right) , \\ \varvec{h}_{v}^{(k)}&=\text {UPDATE}^{(k)}\left( \varvec{h}_{v}^{(k-1)}, \varvec{a}_{v}^{(k)} \right) . \end{aligned} \end{aligned}$$

where ${\mathcal {N}}(v)$ is the set of neighbors of node *v*, $\text {AGGREGATE}^{(k)}(\cdot )$ is the aggregation function for gathering neighboring messages for the central node, $\text {UPDATE}^{(k)}(\cdot )$ is the update function for regenerating the node representation. We initialize the node representation at the 0-th layer as the node feature, that is, $\varvec{h}_{v}^{(0)}=\varvec{x}_{v}$. To obtain the graph representation $\varvec{h}_G$, the $\text {READOUT}(\cdot )$ function is adopted to integrate node representation for permutation invariant pooling, such as sum and average:

5 $$\begin{aligned} \varvec{h}_g=\text {READOUT}\left( \varvec{h}_v^{(K)}|v \in {\mathcal {V}} \right) , \end{aligned}$$

where *K* is the number of GNN layers. The graph representation vector $\varvec{h}_g$ can then be used for downstream task prediction. For conciseness, we ignore the superscript (*K*) and denote $\varvec{h}_v$ as the representation of node *v* and denote $\text {GNN}(\cdot )$ as graph neural network hereafter.

### The FREL framework

Following generic “pretrain, fine-tune” pipelines, we first pretrain a simple GNN model with self-supervised objective and then fine-tune it on the downstream molecular property prediction tasks. The core idea of the FREL framework lies in the design of self-supervised objective, which facilitates the learning of underlying biomedical semantics by the model. The overall pretraining process involves two perspectives, known as dual-channel pretraining, which includes contrastive learning and generative learning channel. In the subsequent fine-tuning phase, we take the weights of the learned model and tune it on the labeled datasets with supervised information.

In the following, We first elaborate on the two pretext tasks specialized for molecular fragment and introduce a integrated objective for pretraining. Then, we justify the effectiveness of our pretraining strategy from the perspective of information theory.

#### The contrastive learning channel

Contrary to prior works that generate augmented views from local and global aspects with random perturbation [57–59], our contrastive learning channel takes into consideration the molecular fragment to construct positive pairs.

To be specific, we first leverage the GNN encoder to obtain node embeddings for both views. For the first view, we take the mean of node embeddings belonging to the same fragment as fragment representation. Then, we perform fragment-level self-attention to further capture the correlation between different substructures and make weighted pooling based on attention coefficients. The second view is obtained by simple mean pooling upon node embeddings. Eventually, we employ a contrastive objective to enforce the embedded molecular views agree with each other and can be discriminated from embeddings of other molecules.

In our FREL model, we use BRICS algorithm [60] to decompose the molecule into fragments. We further adopt mean pooling and attentive pooling to get the fragment and molecule representation as:

6 $$\begin{aligned} \varvec{h}_{f}^m = \frac{1}{|{\mathcal {F}}^m|}\sum _{v \in {\mathcal {F}}^m}\varvec{h}_v, \qquad \qquad \widetilde{\varvec{h}}_{g} = \sum _{m \in {\mathcal {M}}}\alpha ^m\varvec{h}_f^m, \end{aligned}$$

where $\varvec{h}_f^{m}, \widetilde{\varvec{h}}_g\in {\mathbb {R}}^{D}$ represent the representation of fragment and molecule, respectively. The ${\mathcal {F}}^m$ is the node set of fragment *m* and scalar value $|{\mathcal {F}}_i|$ is the corresponding number of node. We leverage an self-attention network [61] that learns to adjust the contribution of each fragment and generate a fragment-based molecular representation with weighted coefficients. Formally, the attention coefficient $\alpha ^m$ denoting the contribution of the $m$-th fragment is computed by:

7 $$\begin{aligned} \alpha ^m = \frac{\exp (w^m)}{\sum _{m' \in {\mathcal {M}}} \exp (w^{m'})}\varvec{v}_f^m, \end{aligned}$$

8 $$\begin{aligned} w^m = \frac{1}{|{\mathcal {B}}|} \sum _{\varvec{h}_f^m \in {\mathcal {B}}} \frac{\varvec{q}_f^m\cdot \varvec{k}_f^m}{\sqrt{D}}, \end{aligned}$$

9 $$\begin{aligned} \varvec{q}_f^m=\varvec{h}_f^m\cdot \varvec{W}_q, \qquad \varvec{k}_f^m=\varvec{h}_f^m\cdot \varvec{W}_k, \qquad \varvec{v}_f^m=\varvec{h}_f^m\cdot \varvec{W}_v, \end{aligned}$$

where $\varvec{q}_f^m, \varvec{k}_f^m, \varvec{v}_f^m \in {\mathbb {R}}^{D}$, $\varvec{W}_q,\varvec{W}_k,\varvec{W}_v, \in {\mathbb {R}}^{D\times D}$ are trainable parameters in the attention network, and ${\mathcal {B}}$ denotes the set of molecules in the current training batch. Finally, our contrastive objective aims to align the fragment-based molecular embeddings with atom-based molecular embeddings.

For any molecule $g_i$, we specify the fragment-based embedding $\widetilde{\varvec{h}}_g^i$ as the anchor, while the atom-based embedding $\varvec{h}_g^i$ is regarded as the positive sample. Other generated embeddings $\{\varvec{h}_g^j\}_{i\ne j}$ in the same batch are negative samples. By performing popular and effective Information Noice Contrastive Estimation (InfoNCE) objective as prior studies, the pairwise objective is formalized as follow:

10 $$\begin{aligned} {\mathcal {L}}_{{\mathcal {C}}} =\frac{1}{|{\mathcal {B}}|}\sum _{i\in {\mathcal {B}}}\left[ -\log \frac{\exp (\theta (\widetilde{\varvec{h}}_g^i, \varvec{h}_g^i)/\tau )}{\sum _{j \in {\mathcal {B}}} \exp (\theta (\widetilde{\varvec{h}}_g^i, \varvec{h}_g^j) / \tau )}\right] , \end{aligned}$$

where the critic function $\theta$ computes the similarity score of contrastive pairs and the hyperparameter $\tau$ adjusts the dynamic range to control the smoothness of the distribution. The ${\mathcal {B}}$ denotes the number of training samples in the batch.

#### The generative learning channel

While contrastive learning maximizes the agreement between molecule pairs, generative learning, on the other hand, extracts unsupervised signals from the molecule itself. Prior graph masked auto-encoder (GMAE), which targets reconstructing graph structures and features, are mainly performed on the complete graph for recovery. However, it may undermine the intrinsic information (e.g. acidity and polarity of carboxyl) of molecular fragments when encoded in different molecules, thus impairs the prediction performance.

To this end, we propose to conduct GMAE at fragment level to preserve intrinsic information where possible. Formally, we randomly select partial nodes to constitute a subset $\hat{{\mathcal {V}}}\subset {\mathcal {V}}$ and mask their node features with the special token $[\text {MASK}]$. Given the graph encoder $\text {GNN}_{enc}(\cdot )$, decoder $\text {GNN}_{dec}(\cdot )$ and masked node feature $\hat{\varvec{x}_v}$, the reconstructed node representation $\hat{\varvec{z}_{v}}$is formalized as below:

11 $$\begin{aligned} \hat{\varvec{z}_{v}} = \text {GNN}_{dec}\left( \text {GNN}_{enc}(\hat{\varvec{x}_v}, \varvec{x}_{uv})\right) \end{aligned}$$

Following GraphMAE [27], we leverage scaled cosine error as the criterion to mitigate the *sensitivity* and *low selectivity* problem [62]. The generative loss is defined as:

12 $$\begin{aligned} {\mathcal {L}}_{{\mathcal {G}}}=\frac{1}{|{\mathcal {B}}|} \sum _{i\in {\mathcal {B}}}\left[ \frac{1}{|\hat{{\mathcal {V}}_i}|}\sum _{v\in \hat{{\mathcal {V}}_i}} {\left( 1-\frac{{\varvec{x}_v}^T\hat{\varvec{z}_v}}{\Vert \varvec{x}_v\Vert \cdot \Vert \hat{\varvec{z}_v} \Vert } \right) }^{\gamma } \right] , \quad \gamma \ge 1 \end{aligned}$$

where the scaling factor $\gamma$ is a self-defined hyper-parameter. It is worth noting that the graph encoder and decoder are both performed at fragment level rather than graph level. By now, the overall pretraining objective can be summarized as follow:

13 $$\begin{aligned} {\mathcal {L}}={\mathcal {L}}_{{\mathcal {C}}} + {\mathcal {L}}_{{\mathcal {G}}} \end{aligned}$$

#### Theoretical analysis

Recalling that the overall optimization objective has the form of the summation of contrastive loss and generative loss. In this section, we further provide a deeper insight into the theoretical support of our optimization objective. We demonstrate that minimizing the total loss ${\mathcal {L}}$ is equivalent to maximizing a lower bound on the sum of two types of mutual information. The first type is based on the mutual information between positive pairs in contrastive learning, while the second type is based on the mutual information between the original input and the encoded representation in generative learning. To be specific, we propose the following theorem:

##### Theorem 1

*The sum of mutual information can be lower bounded by*

14 $$\begin{aligned} I(\widetilde{\varvec{h}}_g^i,\varvec{h}_g^i) + I(\varvec{x}, \hat{\varvec{z}}) \ge -{\mathcal {L}} + \text {const} \end{aligned}$$

where $I(\widetilde{\varvec{h}}_g^i,\varvec{h}_g^i)$ is the mutual information between positive pair in contrastive learning, and $I(\varvec{x}, \hat{\varvec{z}})$ is the mutual information between raw input and the encoded representation in generative learning. The detailed proof is included in the Additional file 4.

Intuitively, the result of theoretical analysis tells us that with the continuous optimization of model parameters, on the one hand, contrastive learning improves the mutual information among positive samples by comparing them with negative samples, which learns the agreement between molecules; generative learning, on the other hand, improves the mutual information between the original input and the encoded representation by reconstruction loss, which learns the agreement within molecules. Overall, by constructing high-quality contrastive samples and obtaining better masked representations with molecular fragments, we simultaneously learn the intra- and inter-molecule agreement, which is the key to the effectiveness of our proposed model FREL.

### More detailed experimental configurations

#### Datasets

For fair comparison with the other pre-trained models, we choose GEOM-Drugs [39] as the pre-training dataset, which contains 304,466 mid-sized organic molecules with experimental data. Due to the limitation of computing resources, we follow GraphMVP [20] to sample a subset of 50K molecules for practical training. We then conducted fine-tuning using various datasets, sourced from MoleculeNet [10] and ChEMBL [48]. The datasets used in fine-tuning encompass a broad range of applications including both biological and pharmaceutical tasks.These properties can be divided into three categories: physical chemistry, biophysics, physiology. Basic dataset statistics is summarized in Table 4.

**Table 4:** Statistics of datasets used in experiments. The first section describes the datasets used for pre-training; the later two sections describe datasets for fine-tuning

	Dataset	#Molecules	Avg. #atoms	Avg. #bonds	#Tasks	Avg. degree
	GEOM-Drug	304,466	44.40	46.40	–	2.09
Classification	BBBP	2,039	24.06	25.95	1	2.16
	Tox21	7,831	18.57	19.29	12	2.08
	ToxCast	8,576	18.78	19.26	617	2.05
	SIDER	1,427	33.64	35.36	27	2.10
	MUV	93,087	24.23	26.28	17	2.17
	HIV	41,127	25.51	27.47	1	2.15
	BACE	1,513	34.09	36.86	1	2.16
Reg.	ESOL	1,128	13.30	13.69	1	2.06
	Lipophilicity	4,200	27.04	29.50	1	2.18
	Malaria	9,999	30.36	33.20	1	2.19

- Physical chemistry.

The ESOL dataset [47] contains data on the solubility of molecules in water. Similarly, the Lipophilicity dataset represents a subset of the ChEMBL database [48] and records data on the octanol/water distribution coefficient of molecules.

- Biophysics.

The HIV dataset (AIDS Antiviral Screen) [45] was developed by the Drug Therapeutics Program (DTP), and is designed to evaluate the ability of molecular compounds to inhibit HIV replication. The Maximum Unbiased Validation (MUV) group [44] was selected from PubChem BioAssay via a refined nearest neighbor analysis approach. The BACE dataset, on the other hand, offers qualitative binding data on a collection of inhibitors of human $\beta$-secretase 1 (BACE-1) [46]. The Malaria dataset [25] gauges drug efficacy in inhibiting parasites responsible for causing malaria.

- Physiology.

The Blood-brain barrier penetration (BBBP) dataset [40] models the barrier permeability of molecules targeting central nervous system. Tox21 [41] and ToxCast [42] are all related to the toxicity of molecular compounds. The Side Effect Resource (SIDER) [43] is a dataset measuring the adverse drug reactions of 27 system organ classes of marketed drugs.

For those datasets for fine-tuning, we follow OGB [15] that uses scaffolds to split training/test/validation subsets with a split ratio of 80%/10%/10%. The scaffold split constructs the out-of-distribution scenario, which is more in line with the actual drug development situation.

#### Baselines

For comprehensive comparison, we select the following two groups of Self-Supervised Learning (SSL) methods as primary baselines in our experiments.

- Generic graph SSL models: AttrMask, ContextPred [16], InfoGraph [49], GPT-GNN [26], GraphLoG [18], GraphCL [17], JOAO [28], and GraphMAE [27].
- Molecular SSL models: GROVER-Contextual (GROVER-C), GROVER-Motif (GROVER-M) [34], and GraphMVP [20].

In the pretraining stage, all the above SSL approaches are trained on the same dataset based on GEOM-Drugs. We also report performance with a randomly initialized model as the non-pretraining baseline. To ensure the performance is comparable with existing work, we report most of baseline performance from previously published results [20]. However, we reproduce the performance of GraphMAE [27] and report the corresponding results to avoid inconsistent comparison with different pre-training dataset.

#### Implementation details

All of the experiments are deployed on a computer server with 4 NVIDIA GeForce RTX 3090 GPUs (with 24GB memory each) and 256 AMD EPYC 7742 CPUs. We adopt Glorot initialization [63] for the initialization of the model parameters and the Adam optimizer [64] for optimization.

In the selection of the pre-training backbone model, all of the baseline methods and our model follow the widely-used settings proposed by Hu et al. [16]. On one hand, they have demonstrated that the GIN model exhibits significant benefits in pre-training while maintaining a moderate parameter size. On the other hand, considering that the baselines we compare against employ the GIN model as the backbone, we maintain consistent experimental settings to ensure a fair comparison. Next, we provide a detailed description of the hyperparameter settings for the models. For the GNN encoder, we follow widely-used settings [16], where the network consists of 5 layers and the number of neurons in the hidden layers is set to 300. The dropout ratio is set to 0 in the pre-training phase and 0.5 in all downstream tasks. The GNN decoder in our framework follows the setting of GraphMAE, which utilizes a single-layer GIN as its decoder. This choice is based on the claim made by Hou et al. [27] that a GNN decoder can reconstruct the input features of a node using a set of neighboring nodes, rather than relying solely on the node itself.

For other hyperparameters used in model training, we follow the settings used in GraphMVP [20] and assure that all of the baselines align with this setting for fair comparison. To be specific, we set the batch size to 256, and the learning rate for both upstream and downstream models is set to 0.001. We also set the number of workers to 8 to reduce training time. Moreover, the initialization random seed used in pretraining is fixed to 42. For downstream evaluation, we randomly run the same scaffold split on each dataset three times with different seeds, which also align with the settings in GraphMVP. Note that the temperature coefficient $\tau$ for contrastive learning and the mask ratio *m* for generative learning are two highly relevant parameters for the performance of FREL. We conduct detailed discussions and experimental explorations on these parameters in the sensitivity analysis section. The source code of our experiment is available at https://github.com/Ruowu9944/FREL.

## Supplementary information

**Additional file 1.** Detailed description of exploratory experiment.

**Additional file 2.** Ablation study with different channel.

**Additional file 3.** Performance variation with hyper-parameter *m* and $\tau$.

**Additional file 4.** Proof of Theorem 1.

## Data Availability

The GEOM-Drugs dataset is available at https://dataverse.harvard.edu/api/access/datafile/4327252. The datasets provided by MoleculeNet are available at https://github.com/deepchem/deepchem. The malaria dataset is available at https://raw.githubusercontent.com/HIPS/neural-fingerprint/master/data/2015-06-03-malaria/malaria-processed.csv. Our code is available at https://github.com/Ruowu9944/FREL.

## Abbreviations

FPR: False positive rate
TPR: True positive rate
ROC: Receiver operating characteristic
ROC-AUC: The area under the ROC-curve
RMSE: Root mean squared error
SOTA: State-of-the-art
FREL: FRagment-based dual-channEL pretraining
GNN: Graph neural network
SSL: Self-supervised learning
GMAE: Graph masked auto-encoder
