# Supplementary material for: Enhancing drug property prediction with dual-channel transfer learning based on molecular fragment
Source: BMC Bioinformatics. 2023 Jul 21;24:293. doi: 10.1186/s12859-023-05413-x (PMC10360281; doi:10.1186/s12859-023-05413-x)
Supplement: Supplementary file 1 — Additional file 1. Detailed description of exploratory experiment. [file 12859_2023_5413_MOESM1_ESM.pdf]

ADDITIONAL FILE

Proof of Theorem 1 for Enhancing Drug Property Prediction with Dual-Channel Transfer Learning based on Molecular Fragment

Yue Wu[1], Xinran Ni[2], Zhihao Wang[3] and Weike Feng[1*]

[*]Correspondence:
fengweike315@163.com
[1]College of Traditional Chinese Medicine, Shandong University of Traditional Chinese Medicine, Jinan, CN
Full list of author information is available at the end of the article

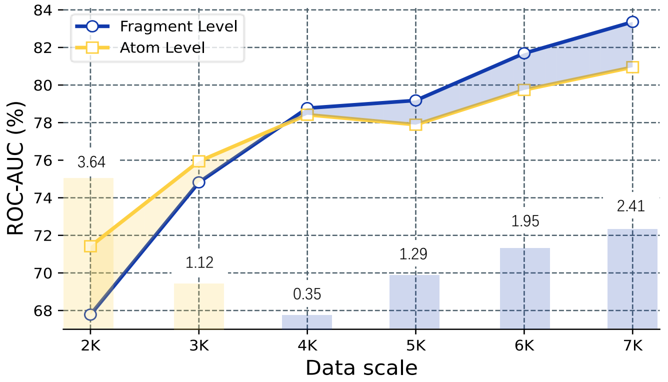

Figure 1 Exploratory Experiment for the feasibility of fragment-based model design.

We present a exploratory experiment to verify the feasibility and effectiveness of fragment-based model design apart from intuitive inspiration. We design two self-attention models based on **atom-level** and **fragment-level** information respectively to compare the respective performance and the results are shown in the Figure 1. The line chart reflects the performance variation, where blue line represents fragment-level attention and yellow line represents the atom-level attention. We also use bar chart to demonstrate the performance gap and the color of bar indicates which strategy is dominant.

Specifically, we randomly select 2K/4K/8K/16K/32K molecules from HIV dataset [1] for training. We adopt a widely-used Graph Isomorphism Network(GIN)[2], which receives as input the graph adjacency matrix and attributes of atoms and bonds, to produce the embedding vectors. We further perform a **atom-level** and **fragment-level** self-attention to compare their performance. The line chart reflects the performance variation, where blue line represents fragment-level attention and yellow line represents the atom-level attention. We also use bar chart to demonstrate the performance gap and the color of bar indicates which strategy is dominant.We report the classification performance in terms of the Area Under the ROC-Curve (ROC-AUC), where higher values indicate better performance.

With the same model capacity, it can be observed that the fragment-based model gradually catches up and overtakes the atom-level model. Moreover, the performance gap becomes larger with the increase of data size. It further motivates us

to explore useful fragment-based pretraining framework to aid molecular property prediction.

Author details
[1]College of Traditional Chinese Medicine, Shandong University of Traditional Chinese Medicine, Jinan, CN.
[2]College of Pharmacy, Shandong University of Traditional Chinese Medicine, Jinan, CN. [3]College of Intelligence and Information Engineering, Shandong University of Traditional Chinese Medicine, Jinan, CN.

References

1. AIDS Antiviral Screen Data. `https://wiki.nci.nih.gov/display/NCIDTPdata/AIDS+Antiviral+Screen+Data`
2. Xu, K., Hu, W., Leskovec, J., Jegelka, S.: How Powerful are Graph Neural Networks? In: ICLR (2019)
