# Supplementary material for: Enhancing drug property prediction with dual-channel transfer learning based on molecular fragment
Source: BMC Bioinformatics. 2023 Jul 21;24:293. doi: 10.1186/s12859-023-05413-x (PMC10360281; doi:10.1186/s12859-023-05413-x)
Supplement: Supplementary file 2 — Additional file 2. Ablation study with different channel. [file 12859_2023_5413_MOESM2_ESM.pdf]

Ablation study

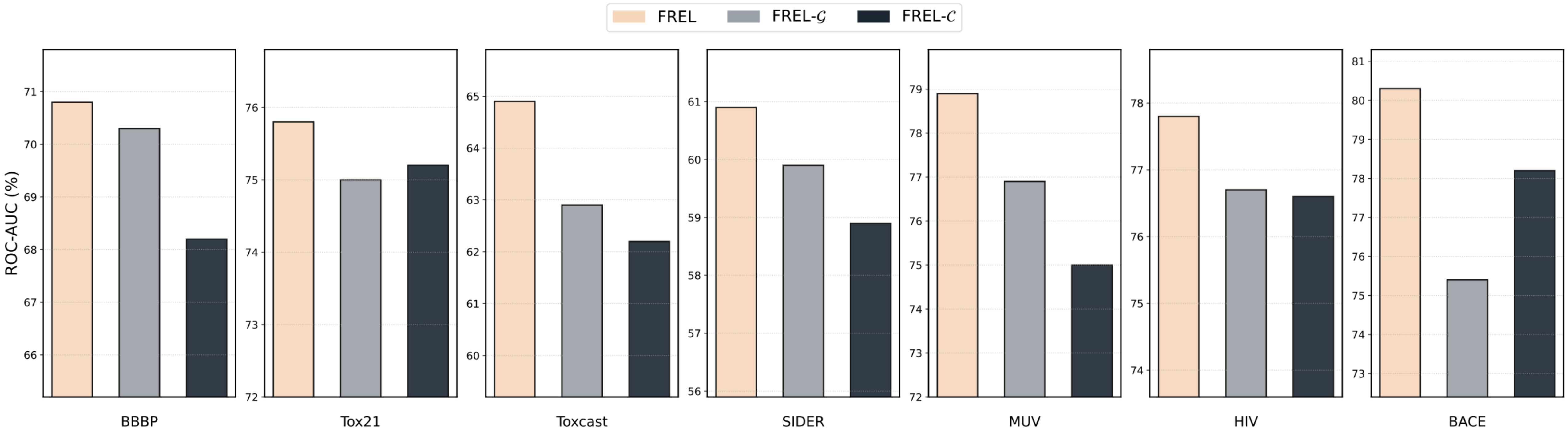

Figure. The ablation study with different channels on seven classification tasks.
