# Supplementary material for: Enhancing drug property prediction with dual-channel transfer learning based on molecular fragment
Source: BMC Bioinformatics. 2023 Jul 21;24:293. doi: 10.1186/s12859-023-05413-x (PMC10360281; doi:10.1186/s12859-023-05413-x)
Supplement: Supplementary file 3 — Additional file 3. Performance variation with hyper-parameter m and $\tau$τ. [file 12859_2023_5413_MOESM3_ESM.pdf]

Sensitivity Analysis

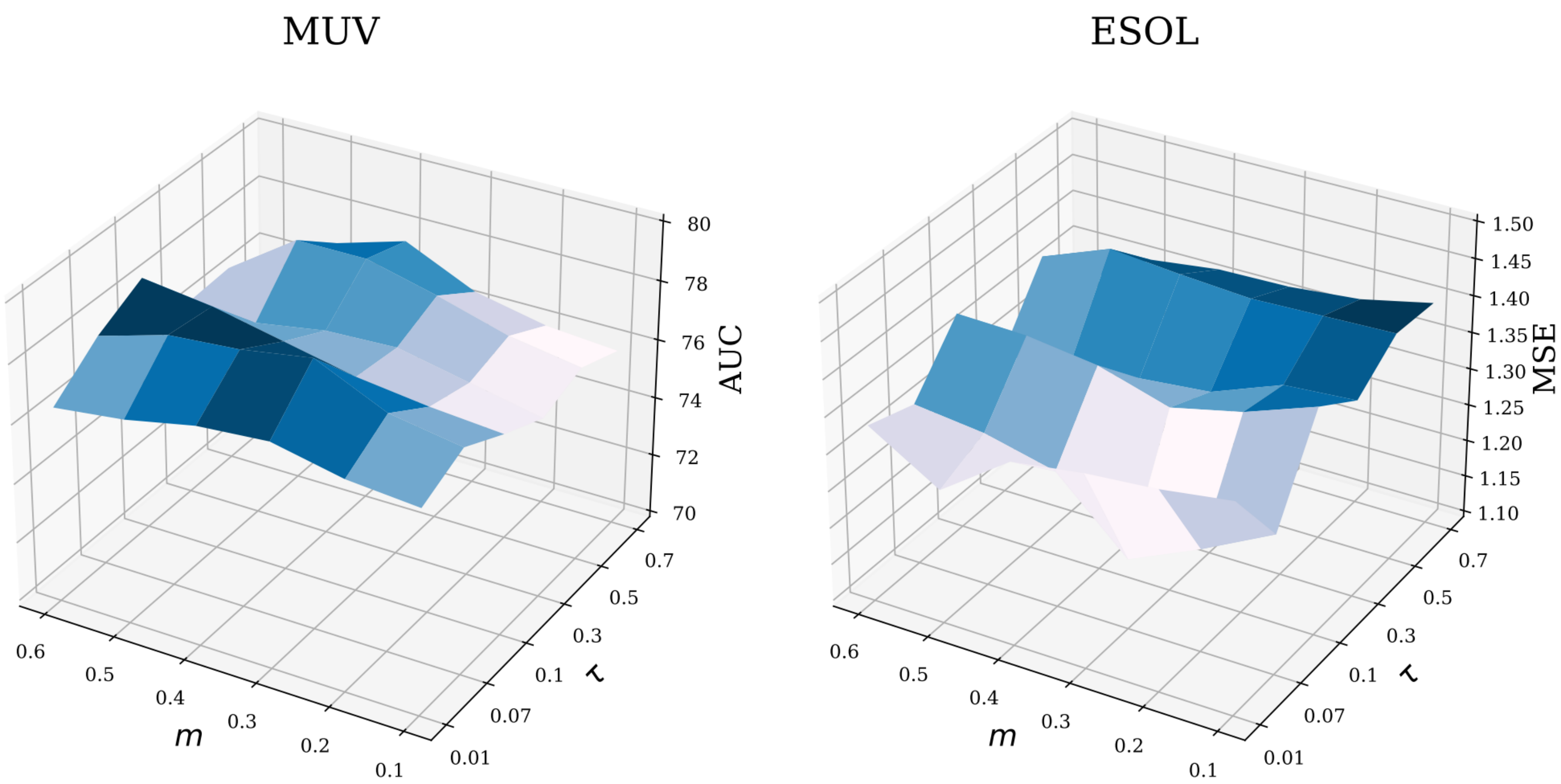

Figure. Performance variation with hyper-parameter m and τ on MUV and ESOL datasets.
