# Supplementary material for: Enhancing drug property prediction with dual-channel transfer learning based on molecular fragment
Source: BMC Bioinformatics. 2023 Jul 21;24:293. doi: 10.1186/s12859-023-05413-x (PMC10360281; doi:10.1186/s12859-023-05413-x)
Supplement: Supplementary file 4 — Additional file 4. Proof of Theorem 1. [file 12859_2023_5413_MOESM4_ESM.pdf]

ADDITIONAL FILE

Proof of Theorem 1 for Enhancing Drug Property Prediction with Dual-Channel Transfer Learning based on Molecular Fragment

Yue Wu[1], Xinran Ni[2], Zhihao Wang[3] and Weike Feng[1*]

*Correspondence: fengweike315@163.com
[1]College of Traditional Chinese Medicine, Shandong University of Traditional Chinese Medicine, Jinan, CN
Full list of author information is available at the end of the article

Theorem 1: *The sum of mutual information can be lower bounded by*

$$I(\widetilde{\boldsymbol{h}}_g^i, \boldsymbol{h}_g^i) + I(\boldsymbol{x}, \hat{\boldsymbol{z}}) \geq -\mathcal{L} + \text{const} \tag{1}$$

where $I(\widetilde{\boldsymbol{h}}_g^i, \boldsymbol{h}_g^i)$ is the mutual information between positive pair in contrastive learning, and $I(\boldsymbol{x}, \hat{\boldsymbol{z}})$ is the mutual information between raw input and the encoded representation in generative learning.

$$I(\widetilde{\boldsymbol{h}}_g^i, \boldsymbol{h}_g^i) + I(\boldsymbol{x}, \hat{\boldsymbol{z}}) \geq -\mathcal{L} + \text{const} \tag{2}$$

where $I(\widetilde{\boldsymbol{h}}_g^i, \boldsymbol{h}_g^i)$ is the mutual information between positive pair in contrastive learning, and $I(\boldsymbol{x}, \hat{\boldsymbol{z}})$ is the mutual information between raw input and the encoded representation in generative learning.

Proof For simplicity of notation, we use term $I(\boldsymbol{x}_1, \boldsymbol{x}_2)$ to represent $I(\widetilde{\boldsymbol{h}}_g^i, \boldsymbol{h}_g^i)$ in the following derivation. According to previous research [1], it is proved that expression $\exp(\theta(\boldsymbol{x}_1, \boldsymbol{x}_2))$ is proportional to $\frac{p(\boldsymbol{x}_1|\boldsymbol{x}_2)}{\boldsymbol{x}_1)}$. Thus,

$$\mathcal{L}_{\mathcal{C}} = -\mathbb{E}_{\boldsymbol{X}} \log \left[\frac{\frac{p(\boldsymbol{x}_1|\boldsymbol{x}_2)}{p(\boldsymbol{x}_1)}}{\frac{p(\boldsymbol{x}_1|\boldsymbol{x}_2)}{p(\boldsymbol{x}_1)} + \sum_{\boldsymbol{x}_j \in \boldsymbol{X}_{neg}} \frac{p(\boldsymbol{x}_j|\boldsymbol{x}_2)}{p(\boldsymbol{x}_j)}} \right], \tag{3}$$

$$= \mathbb{E}_{\boldsymbol{X}} \log \left[1 + \frac{p(\boldsymbol{x}_1)}{p(\boldsymbol{x}_1|\boldsymbol{x}_2)} \sum_{\boldsymbol{x}_j \in \boldsymbol{X}_{neg}} \frac{p(\boldsymbol{x}_j|\boldsymbol{x}_2)}{p(\boldsymbol{x}_j)} \right], \tag{4}$$

$$\approx \mathbb{E}_{\boldsymbol{X}} \log \left[1 + \frac{p(\boldsymbol{x}_1)}{p(\boldsymbol{x}_1|\boldsymbol{x}_2)} (|\mathcal{B}| - 1) \mathbb{E}_{\boldsymbol{x}_j} \frac{p(\boldsymbol{x}_j|\boldsymbol{x}_2)}{p(\boldsymbol{x}_j)} \right], \tag{5}$$

$$= \mathbb{E}_{\boldsymbol{X}} \log \left[1 + \frac{p(\boldsymbol{x}_1)}{p(\boldsymbol{x}_1|\boldsymbol{x}_2)} (|\mathcal{B}| - 1) \right], \tag{6}$$

$$\geq \mathbb{E}_{\boldsymbol{X}} \log \left[\frac{p(\boldsymbol{x}_1)}{p(\boldsymbol{x}_1|\boldsymbol{x}_2)} |\mathcal{B}| \right], \tag{7}$$

$$= -I(\boldsymbol{x}_1, \boldsymbol{x}_2) + \log(|\mathcal{B}|) \tag{8}$$

For generative loss, minimizing the reconstruction loss is proved to be equivalent to maximize a lower bound of the mutual information between the raw input $\boldsymbol{x}$ and

encoded representation $\hat{\boldsymbol{z}}$ [2], which can be formalized as:

$$\mathcal{L}_{\mathcal{G}} \propto \mathbb{E}_{p(\boldsymbol{x}_v,\hat{\boldsymbol{z}_v})}\left[\|\boldsymbol{x}_v - \hat{\boldsymbol{z}_v}\|_2^2\right] \tag{9}$$

$$\geq -\mathbb{E}_{p(\boldsymbol{x}_v,\hat{\boldsymbol{z}_v})} log \frac{p(\boldsymbol{x}_v|\hat{\boldsymbol{z}_v})}{p(\boldsymbol{x}_v)} + H(\boldsymbol{x}) \tag{10}$$

$$= -I(\boldsymbol{x},\hat{\boldsymbol{z}}) + H(\boldsymbol{x}) \tag{11}$$

where $H(\boldsymbol{x})$ is the constant entropy of $\boldsymbol{x}$. Combining Equation (8) and Equation (11), we arrive at the main theorem.

$$I(\widetilde{\boldsymbol{h}}_g^i, \boldsymbol{h}_g^i) + I(\boldsymbol{x},\hat{\boldsymbol{z}}) \geq -(\mathcal{L}_{\mathcal{C}} + \mathcal{L}_{\mathcal{G}}) + \log(|\mathcal{B}|) + H(\boldsymbol{x}) \tag{12}$$

$$= -\mathcal{L} + \text{const} \tag{13}$$

Author details
[1]College of Traditional Chinese Medicine, Shandong University of Traditional Chinese Medicine, Jinan, CN. [2]College of Pharmacy, Shandong University of Traditional Chinese Medicine, Jinan, CN. [3]College of Intelligence and Information Engineering, Shandong University of Traditional Chinese Medicine, Jinan, CN.

References

1. Oord, A.v.d., Li, Y., Vinyals, O.: Representation learning with contrastive predictive coding. arXiv preprint arXiv:1807.03748 (2018)
2. Vincent, P., Larochelle, H., Lajoie, I., Bengio, Y., Manzagol, P.-A., Bottou, L.: Stacked denoising autoencoders: Learning useful representations in a deep network with a local denoising criterion. Journal of machine learning research **11**(12) (2010)
